# Case Report: Severe adolescent-onset schizophrenia with Capgras syndrome and remission during clozapine-centered treatment after earthquake-related displacement and peer victimization

**DOI:** 10.3389/fpsyt.2026.1887119

**Published:** 2026-07-28

**Authors:** Mustafa Çobaner, Ali Evren Tufan

**Affiliations:** 1 Department of Child and Adolescent Psychiatry, University of Health Sciences, Trabzon Kanuni Training and Research Hospital, Trabzon, Türkiye; 2 Department of Child and Adolescent Psychiatry, Bolu Abant İzzet Baysal University Faculty of Medicine, Bolu, Türkiye

**Keywords:** adolescent, Capgras syndrome, case report, clozapine, delusional misidentification, first-episode psychosis, peer victimization, schizophrenia

## Abstract

Adolescent first-episode psychosis may combine schizophrenia-spectrum, affective, trauma-related, and developmental features, and Capgras syndrome is uncommon in this age group. We report a confidentially de-identified case of a 16-year-old female high-school student with no previous psychiatric admission or known substance use who developed severe insomnia, reduced self-care, external auditory hallucinations, paranoid and referential ideation, Capgras-type misidentification of her father, and a multithemed bizarre delusional system after cumulative psychosocial stressors including earthquake-related displacement, relocation, school transition, peer exclusion, appearance-related mockery, and family-school conflict. Laboratory tests, brain magnetic resonance imaging, electroencephalography, substance screening, and child neurology consultation were unrevealing. DSM-5-TR schizophreniform disorder was the initial working diagnosis; bipolar disorder with psychotic features, schizoaffective disorder, brief psychotic disorder, trauma-related or dissociative phenomena, substance/medication-induced psychosis, autoimmune encephalitis, and temporal lobe epilepsy were considered. Risperidone- and olanzapine-based treatment produced partial but insufficient improvement. Clozapine was initiated at admission week 8 and titrated to 175 mg/day over approximately 4 weeks. Marked improvement was observed by week 2 of clozapine-centered treatment and remission by week 4. At admission week 12, the Clinical Global Impression-Severity score was 1, the Positive and Negative Syndrome Scale score was 45, and the Young Mania Rating Scale score was 4. At 8-month follow-up, the patient remained clinically stable on clozapine 175 mg/day, and the diagnosis was maintained as schizophrenia because the longitudinal course exceeded 6 months. This case highlights the diagnostic complexity of adolescent-onset psychosis with Capgras syndrome, the need to distinguish contextual stressors from primary etiology, and the cautious, monitored consideration of clozapine when severe schizophrenia-spectrum psychosis shows insufficient response to standard antipsychotic trials.

## Introduction

This case report uses DSM-5-TR terminology and follows CARE case-report principles (1–3). Early-onset psychosis can interrupt neurodevelopmental, academic, peer, and family functioning during a sensitive developmental period. In adolescents, first presentations may be diagnostically complex because hallucinations and delusions can coexist with sleep disruption, affective arousal, trauma-related hypervigilance, dissociation, developmental vulnerability, and identity or body-image concerns. Accurate formulation therefore requires careful phenomenological assessment, exclusion of medical and neurological causes, and longitudinal follow-up when differentiating schizophreniform disorder, schizophrenia, bipolar disorder with psychotic features, schizoaffective disorder, brief psychotic disorder, substance/medication-induced psychosis, and psychosis secondary to neurological or medical conditions (1, 4–8).

Psychosocial adversity, including trauma exposure, displacement or migration-related stress, social exclusion, and peer victimization, is associated with psychosis risk and psychotic experiences in epidemiological literature (9–11). In an individual case, however, such associations should be interpreted within a stress-diathesis framework rather than as evidence of direct causality. This distinction is especially important in adolescent practice: psychosocial context should inform care and safety planning without obscuring a primary psychotic disorder when fixed delusions, external hallucinations, disorganization, absent insight, and functional decline are present.

Delusional misidentification syndromes, including Capgras syndrome, involve the belief that a familiar person has been replaced by an identical impostor. Pediatric Capgras syndrome is rare and has been reported with schizophrenia-spectrum disorders, mood disorders, obsessive-compulsive disorder, trauma-related presentations, and neurological conditions (12–18). Recent pediatric reports also emphasize that treatment may require more than antipsychotic monotherapy, including structured family work, exposure-based approaches when obsessive-compulsive symptoms are prominent, psychoeducation, and safety planning (13, 16, 17). Capgras phenomena can carry clinical risk because fear or hostility may become directed toward the misidentified person, so direct assessment of risk, family safety, and the need for supervised contact is warranted (14, 18, 19).

This case contributes to the limited pediatric Capgras literature by bringing together severe adolescent-onset schizophrenia-spectrum psychosis, Capgras-type misidentification of a parent, cumulative displacement and peer-victimization stressors, selected longitudinal rating-scale documentation, insufficient response to two antipsychotic trials, and carefully monitored remission during clozapine-centered treatment. The contribution lies primarily in this clinical constellation rather than in any single feature alone. The report also illustrates how longitudinal assessment supported schizophrenia after an initial schizophreniform diagnosis, while the clozapine response remained clinically important but not causally definitive.

## Case description

### Patient information

The patient was a 16-year-old female adolescent and 11th-grade high-school student who was the eldest of three siblings and lived with both parents and siblings in a nuclear family. She had no previous psychiatric admission and no known substance use. Exact dates, place names, school identifiers, and unnecessary location details were omitted to protect confidentiality.

The pregnancy was planned and wanted. She was born at term by normal vaginal delivery in a hospital setting. No major prenatal, perinatal, neonatal, developmental, neurodevelopmental, or behavioral problems were reported. Premorbidly, she was described as calm, responsible, academically oriented, initially shy in unfamiliar settings, and sensitive to external evaluation and peer criticism.

Approximately 1 year before presentation, the family had lived in an earthquake-affected city and their home reportedly sustained moderate-to-severe damage. She was not trapped under rubble, and no close family bereavement was reported. After temporary displacement, the family relocated again because of parental work assignments. Approximately 9 months before presentation, she entered a high-achieving academic environment in a new city. Peer exclusion, difficulty making friends, appearance-related mockery, class change after family-school meetings, and escalating family-school conflict were reported in the months before admission. The mother had post-earthquake post-traumatic stress disorder and generalized anxiety disorder treated with citalopram 20 mg/day. No psychotic disorder, schizophrenia, bipolar disorder, epilepsy, suicide attempt, or substance use disorder was reported in first- or second-degree relatives.

### Clinical findings

The patient was brought to emergency care because of acute behavioral deterioration. During the 7–10 days before emergency presentation, insomnia, suspiciousness, reduced self-care, and functional withdrawal escalated; in the final 1–2 days, external auditory hallucinations and the Capgras-type belief that her father had been replaced by an identical twin became explicit. She also developed fixed beliefs that peers had secretly entered the home, stolen intimate belongings, installed hidden cameras, and were talking about her.

At admission, consciousness was clear and orientation was intact. She was guarded and suspicious, with markedly reduced self-care, decreased speech quantity and rate, long response latency, tangential thought process, and loosening of associations. Thought content included persecutory and referential delusions, Capgras-type misidentification, fantastical/religious and mythological delusional themes, grandiose content, erotomanic delusions involving a male peer, somatic delusions concerning body asymmetry, head shape, digestion, and abdominal bloating, and passivity/control delusions involving external control. Affect was blunted and restricted; mood was dysphoric with intermittent irritability. Attention and concentration were impaired, memory was grossly intact, insight was absent, and judgment and reality testing were impaired. Sleep was severely reduced, appetite was decreased, and psychomotor activity included purposeless pacing. Mild libido-related content was documented but interpreted primarily within the erotomanic delusional system rather than as sufficient evidence of mania.

Baseline Clinical Global Impression-Severity (CGI-S) was 7, Positive and Negative Syndrome Scale (PANSS) total was 141 (positive 35, negative 30, general psychopathology 76), and Young Mania Rating Scale (YMRS) was 22. The YMRS elevation was interpreted cautiously because reduced sleep, activation, irritability, and libido-related content were present, but a sustained syndromal manic episode was not established (20–22).

### Timeline

The CARE timeline, medication sequence, selected rating-scale assessment points, and clozapine safety monitoring are summarized in **Tables 1**–**4**. **Table 1** serves as the required clinical timeline display item for this case report.

**Table 1:** Clinical timeline.

Time point	Clinical status	Medication/treatment	Rating scores (key points)	Clinical interpretation
Admission/baseline	Emergency presentation after 7-10 days of worsening insomnia, suspiciousness, reduced self-care, and withdrawal; final 1-2 days included external auditory hallucinations and explicit Capgras-type misidentification of father.	Risperidone 2 mg/day; valproate 250 mg/day; lorazepam 1.25 mg/day as needed. Week 1: risperidone to 4 mg/day and valproate to 750 mg/day.	CGI-S 7; PANSS 141 (P35, N30, G76); YMRS 22.	Very severe first-episode psychosis; YMRS elevation interpreted without sustained syndromal mania.
Week 2	No meaningful improvement; symptom burden increased.	Risperidone/valproate continued/titrated; lorazepam as needed.	Not shown	Persistent severe psychosis despite initial treatment.
Week 4	Ongoing intense delusions and intermittent disorganized behavior.	Risperidone 6 mg/day; valproate continued; olanzapine 2.5 mg/day added; sertraline 25 mg/day added for severe anxiety/ruminative trauma-related distress with mood monitoring.	Not shown	Insufficient response; second antipsychotic and cautious SSRI added.
Week 6	Partial improvement: fewer hallucinations and less delusional preoccupation; residual paranoid/referential ideas, Capgras-related mistrust, and withdrawal persisted.	Risperidone 6 mg/day; olanzapine 20 mg/day; valproate 1500 mg/day; sertraline 100 mg/day.	Not shown	Partial but incomplete response.
Week 8	Partial improvement continued but remission was not achieved; residual delusions and functional impairment persisted.	Risperidone 6 mg/day; olanzapine up to 30 mg/day; valproate 1500 mg/day; sertraline 100 mg/day.	CGI-S approximately 5; PANSS 88 (P19, N21, G48); YMRS 15.	Insufficient response despite high-dose antipsychotic treatment.
Week 8: clozapine initiation	Persistent severe schizophrenia-spectrum psychosis and operational insufficient response after two antipsychotic trials.	Clozapine 25 mg/day started; increased by 12.5 mg/day as tolerated toward 175 mg/day over approximately 4 weeks. Olanzapine reduced by 50% on clozapine day 7 and stopped by day 14; risperidone taper planned later.	No additional score at exact clozapine start beyond week-8 ratings.	Clozapine-centered treatment initiated with goal to avoid long-term antipsychotic polypharmacy.
Admission week 12/clozapine week approximately 4	Marked reduction by clozapine week 2 and substantial remission from week 3 onward; psychotic symptoms considered in remission.	Clozapine 175 mg/day; continued cross-titration strategy for other agents as clinically feasible.	CGI-S 1; PANSS 45 (P10, N15, G20); YMRS 4.	Clinical remission during clozapine-centered treatment.
8-month outpatient follow-up	Clinically stable without relapse; longitudinal diagnosis maintained as schizophrenia because course exceeded 6 months.	Clozapine 175 mg/day. No additional structured rating-scale scores available.	Not available.	Stable maintenance on clozapine; continued follow-up required.

CGI-S, Clinical Global Impression-Severity; PANSS, Positive and Negative Syndrome Scale; YMRS, Young Mania Rating Scale.

**Table 2:** Differential diagnosis and rationale.

Diagnosis considered	Supporting features	Features against/limiting considerations	Final interpretation
Schizophreniform disorder	First psychotic episode; hallucinations; multiple fixed delusions; disorganized thought; functional decline; duration initially less than 6 months.	Longitudinal course exceeded 6 months at follow-up.	Initial working diagnosis; not maintained after longitudinal course exceeded 6 months.
Schizophrenia	Schizophrenia-spectrum phenomenology including bizarre delusions, Capgras syndrome, hallucinations, disorganization, impairment, and clinical course exceeding 6 months.	Six-month DSM-5-TR duration criterion was not met at initial assessment.	Longitudinal diagnosis maintained as schizophrenia at 8-month follow-up.
Brief psychotic disorder	Abrupt onset and psychosocial stressors.	Severity, persistence, prolonged inpatient course, incomplete early remission, and later 6-month course argued against a brief self-limited psychosis.	Considered but less favored.
Bipolar disorder with psychotic features	Reduced sleep, activation, irritability, libido-related content, YMRS elevation.	Psychosis was prominent, bizarre, systematized, not clearly mood-congruent, and accompanied by thought disorganization; no sustained syndromal manic episode established.	Less favored; mood symptoms monitored longitudinally.
Schizoaffective disorder	Psychosis with affective symptoms and elevated YMRS.	No sustained syndromal mood episode explained most of the psychotic course.	Less likely; requires longitudinal reassessment.
PTSD/dissociative or trauma-related phenomena	Earthquake-related displacement, relocation, school transition, peer exclusion, appearance-related mockery, possible hyperarousal and anxiety.	Fixed structured delusions, external auditory hallucinations, Capgras misidentification, loosening of associations, absent insight, impaired reality testing, and longitudinal course not fully accounted for by trauma/dissociation alone.	Relevant stress context and potential precipitating/shaping factors; not primary diagnosis.
Substance/medication-induced or organic psychosis	Acute onset and unusual content required exclusion.	Negative substance screen; unrevealing laboratory tests, MRI, EEG, and child neurology consultation.	Less likely with available data.
Autoimmune encephalitis/temporal lobe epilepsy/other neurological disorder	Acute onset and unusual psychotic content; Capgras phenomena can occur with neurological conditions.	No reported seizures, fluctuating consciousness, catatonia, dyskinesia, autonomic instability, MRI/EEG abnormality, or neurological findings.	Not supported currently; further work-up if red flags emerge.

**Table 3:** Selected key rating-scale assessment points.

Time point	CGI-S	PANSS total	PANSS positive	PANSS negative	PANSS general	YMRS	Clinical meaning
Admission/baseline	7	141	35	30	76	22	Very severe illness; psychotic symptoms dominant.
Week 8/immediately before clozapine initiation	5	88	19	21	48	15	Partial but insufficient response immediately before clozapine initiation.
Admission week 12/discharge/clozapine week approximately 4	1	45	10	15	20	4	Clinician-rated remission during clozapine-centered treatment.
8-month outpatient follow-up	Not available	Not available	Not available	Not available	Not available	Not available	Clinically stable without relapse; remained on clozapine 175 mg/day; diagnosis maintained as schizophrenia.

CGI-S, Clinical Global Impression-Severity; PANSS, Positive and Negative Syndrome Scale; YMRS, Young Mania Rating Scale.

**Table 4:** Clozapine safety monitoring parameters across clinical timeline.

Time point	WBC (x10^9^/L)	ANC (x10^9^//L)	CRP (mg/L)	QTc (ECG) (ms)
Baseline (admission)	7.6	4.1	2.1	409
Week 2	8.2	4.4	2.8	412
Week 4	7.6	3.9	4.6	410
Week 6	6.8	3.6	4.8	407
Week 8 (clozapine initiation)	7.2	3.8	2.3	413
Week 12 (approximately clozapine week 4)	7.0	3.7	4.0	411

Reference ranges: WBC, 4.0-10.0 x 10^9^/L; ANC, ≥1.5 x 10^9^/L; CRP, <5 mg/L; QTc, <450 ms.

### Diagnostic assessment

The initial working diagnosis was DSM-5-TR schizophreniform disorder (1). This formulation was supported by a first psychotic episode with external auditory hallucinations, multiple fixed and bizarre delusions, Capgras-type misidentification, disorganized thought process, functional decline, absent insight, and impaired reality testing, while the duration criterion for schizophrenia had not yet been met. At 8-month follow-up, because the longitudinal clinical course had exceeded 6 months and clinically significant psychotic morbidity had persisted across the episode, the diagnosis was maintained as schizophrenia.

Bipolar disorder with psychotic features and schizoaffective disorder were considered because of reduced sleep, activation, irritability, libido-related content, and elevated YMRS. They became progressively less likely because psychosis was prominent, bizarre, multithemed, not clearly mood-congruent, accompanied by thought disorganization, and not temporally explained by a sustained syndromal manic or depressive episode. Brief psychotic disorder was considered because of abrupt onset and psychosocial stressors, but the severity, sustained inpatient course, incomplete early remission, and later 6-month course argued against a brief self-limited psychosis.

Trauma-related and dissociative phenomena were clinically relevant but were not considered the primary explanation. The earthquake-related displacement, relocation, peer exclusion, appearance-related mockery, and family-school conflict were interpreted as contributing stressors that may have shaped the content and timing of symptoms. They did not, by themselves, account for the fixed structured delusions, external auditory hallucinations, Capgras misidentification, loosening of associations, absent insight, impaired reality testing, and longitudinal schizophrenia-spectrum course. The differential diagnosis therefore distinguished trauma-related psychotic experiences, which often require careful evaluation of intrusions, flashbacks, dissociation, and context-bound threat responses, from a primary psychotic syndrome with persistent, bizarre, systematized delusions and disorganization.

Substance/medication-induced or organic psychosis became less likely after negative substance screening, normal complete blood count, routine biochemistry, thyroid function tests, vitamin B12, folate, brain magnetic resonance imaging, electroencephalography, and child neurology consultation. Autoimmune encephalitis and temporal lobe epilepsy were considered because of acute onset and unusual content; available assessment did not support these diagnoses. Additional work-up would be indicated if red flags emerged, including seizures, fluctuating consciousness, delirium, catatonia, dyskinesia, autonomic instability, rapidly progressive cognitive decline, antipsychotic intolerance, or stereotyped episodic phenomena (23).

### Therapeutic intervention

Initial inpatient treatment consisted of risperidone 2 mg/day, valproate 250 mg/day, and lorazepam 1.25 mg/day as needed. During week 1, risperidone was titrated to 4 mg/day and valproate to 750 mg/day as tolerated. Lorazepam provided some sleep benefit, but psychotic symptoms persisted during the early inpatient course.

By week 4, risperidone had been increased to 6 mg/day. Because intense delusions and intermittent disorganized behavior persisted, olanzapine 2.5 mg/day was added partly for sedation and antipsychotic augmentation while a switch strategy was being considered. Sertraline 25 mg/day was initiated for severe anxiety, ruminative distress, and possible trauma-related symptoms only after the clinical team judged that a sustained manic syndrome was not present; valproate was continued, activation was monitored, and the SSRI was not intended as a treatment for psychosis. By week 6, olanzapine was titrated to 20 mg/day, sertraline to 100 mg/day, valproate to 1500 mg/day, and risperidone remained at 6 mg/day. Hallucination frequency and delusional preoccupation decreased, but residual paranoid and referential ideas, Capgras-related mistrust, and social withdrawal persisted.

At week 8, despite risperidone 6 mg/day, olanzapine up to 30 mg/day, valproate 1500 mg/day, and sertraline 100 mg/day, remission had not been achieved; CGI-S was approximately 5, PANSS was 88, and YMRS was 15. The team considered the response insufficient after two antipsychotic trials in a severe schizophrenia-spectrum presentation. Because the diagnosis was still schizophreniform disorder and antipsychotic exposure was shorter than in many adult treatment-resistance criteria, the insufficient response is described operationally rather than as definitive treatment-resistant schizophrenia. Clozapine was considered because severe psychosis, functional impairment, and residual delusions persisted despite risperidone- and olanzapine-based treatment, and because clozapine has evidence for early-onset or treatment-resistant schizophrenia-spectrum illness when standard antipsychotics are inadequate (5, 6, 24–28).

After family discussion and safety counseling, clozapine was initiated at week 8 at 25 mg/day and increased by 12.5 mg/day as tolerated, reaching 175 mg/day over approximately 4 weeks. A cross-titration strategy was used to avoid long-term antipsychotic polypharmacy while reducing destabilization risk: olanzapine was reduced by approximately 50% on clozapine day 7 and discontinued by day 14; risperidone taper was planned subsequently. Hematological, inflammatory, cardiovascular, seizure, sedation, hypersalivation, constipation, and metabolic risks were reviewed before initiation and monitored during treatment.

### Follow-up and outcomes

By the second week of clozapine-centered treatment, delusions and auditory hallucinations had markedly decreased. From the third week onward, psychotic symptoms substantially remitted. At admission week 12, corresponding to approximately week 4 of clozapine treatment, CGI-S was 1, PANSS was 45 (positive 10, negative 15, general psychopathology 20), and YMRS was 4. Psychotic symptoms were considered in remission.

Clozapine treatment was accompanied by regular hematological, inflammatory, metabolic, and cardiovascular monitoring. Baseline white blood cell count and absolute neutrophil count were within normal limits and remained stable. Serial C-reactive protein values showed no clinically significant elevation suggestive of myocarditis, and electrocardiographic QTc values remained within normal limits. Fasting glucose and lipid profile remained within normal ranges during inpatient follow-up. Mild sedation and hypersalivation occurred during early clozapine treatment but did not require dose adjustment or discontinuation. Constipation, seizures, myocarditis, agranulocytosis, clinically significant arrhythmia, and severe metabolic complications were not observed during the inpatient period. At 8-month outpatient follow-up, the patient remained clinically stable without relapse and continued clozapine 175 mg/day. No additional structured rating-scale scores were available for the 8-month visit.

## Discussion

### Contribution to the literature and context

This case contributes to the limited pediatric Capgras literature by presenting an uncommon combination of adolescent-onset schizophrenia-spectrum psychosis, Capgras-type misidentification of a parent, cumulative displacement and peer-victimization stressors, selected CGI-S/PANSS/YMRS documentation, and monitored remission during clozapine-centered treatment after insufficient response to risperidone- and olanzapine-based treatment. Its contribution lies primarily in the combination of these clinical features rather than in any single component.

Prior pediatric Capgras reports include schizophrenia-spectrum, mood, obsessive-compulsive, and organic presentations, emphasizing the need for broad differential diagnosis and individualized multimodal treatment (12, 13, 16, 17). In first-episode psychosis, Capgras syndrome has been described as a clinically important misidentification phenomenon that may coexist with broader delusional systems (15). Systematic reviews of Capgras cases highlight heterogeneity of etiologies, frequent psychiatric comorbidity, and the importance of risk assessment toward the misidentified person (14, 19). In the present case, the misidentified person was the father; therefore, family safety, supervised contact during the acute phase, psychoeducation, and restoration of trust were clinically important alongside medication.

### Diagnostic reasoning and role of stressors

The presentation favored a schizophrenia-spectrum formulation because the core syndrome consisted of external auditory hallucinations, fixed multimodal and bizarre delusions, Capgras misidentification, tangentiality, loosening of associations, absent insight, impaired reality testing, and functional decline. The elevated YMRS score was clinically relevant but did not establish bipolar disorder because the psychosis was not clearly mood-congruent or temporally explained by a sustained manic episode. Similarly, trauma-related and dissociative phenomena were considered, but the longitudinal course, fixed delusional structure, and disorganization made a primary trauma-related explanation less likely.

The psychosocial stressors remained clinically meaningful as potential precipitating or shaping factors within a biopsychosocial formulation. However, the fixed delusional structure, external hallucinations, disorganization, absent insight, impaired reality testing, and longitudinal course supported a primary schizophrenia-spectrum formulation rather than a purely trauma-related explanation.

### Clozapine rationale and causal caution

Clinical improvement after clozapine initiation was notable, but a single case cannot establish causality. Partial improvement had already occurred with risperidone- and olanzapine-based treatment, and inpatient care, concomitant medications, sleep restoration, and natural illness evolution may have contributed.

The clinical implication is that clozapine may be considered cautiously in selected adolescents with severe schizophrenia-spectrum psychosis and persistent impairment after insufficient response to standard antipsychotic trials, provided shared decision-making, gradual titration, adverse-effect counseling, and hematological and cardiometabolic monitoring are in place (5, 6, 24–28).

### Clozapine safety in an adolescent

Potential clozapine adverse effects considered before and during treatment included neutropenia/agranulocytosis, myocarditis, cardiomyopathy, QTc prolongation, orthostatic hypotension, tachycardia, seizures, sedation, hypersalivation, constipation/ileus, weight gain, dyslipidemia, insulin resistance, and rare severe inflammatory or thromboembolic complications. In this patient, baseline and serial WBC/ANC, CRP, ECG/QTc, fasting glucose, lipids, vital signs, seizure history, bowel function, sedation, and hypersalivation were monitored. Mild sedation and hypersalivation occurred; no serious clozapine-related adverse event was observed during inpatient treatment. This monitoring strengthens the safety documentation but does not remove the need for long-term follow-up.

### Practical take-home messages

Clinicians should suspect Capgras syndrome in adolescents when fear, avoidance, hostility, or mistrust is specifically directed toward a familiar person believed to be replaced, duplicated, demonic, robotic, or otherwise not genuine. Assessment should ask directly but calmly about misidentification beliefs, risk toward the misidentified person, family safety, and the broader delusional system.

Organic work-up should be broadened when onset is acute or atypical, when neurological symptoms are present, or when red flags emerge, including seizures, fluctuating consciousness, delirium, catatonia, dyskinesia, autonomic instability, rapidly progressive cognitive decline, antipsychotic intolerance, abnormal neurological examination, or stereotyped episodic phenomena. In the absence of such findings, longitudinal psychiatric reassessment remains essential.

Clozapine consideration is most defensible when severe schizophrenia-spectrum psychosis and functional impairment persist after adequately dosed antipsychotic trials, safety or prolonged hospitalization is a concern, and family participation in monitoring is feasible. The decision should remain individualized and should not be interpreted as proof that clozapine alone caused improvement.

### Strengths and limitations of the case

Strengths include detailed developmental and psychosocial history, structured mental status examination, medical and neurological exclusion, selected CGI-S, PANSS, and YMRS data, a clear medication timeline, documented clozapine safety monitoring, patient perspective, and 8-month outpatient follow-up. Limitations include the single-case design, absence of a controlled treatment comparison, incomplete ability to separate clozapine effects from cumulative treatment, natural recovery, inpatient containment, and concomitant medications, absence of additional structured rating-scale data at 8-month follow-up, limited information on longer-term school reintegration and functioning, and the need for continued follow-up to assess long-term prognosis.

## Conclusion

Adolescent first-episode psychosis can present with complex, bizarre, and multithemed delusions, including Capgras syndrome. Psychosocial adversity may be clinically relevant but should be interpreted cautiously within a stress-diathesis framework rather than as a sole etiological explanation. In severe schizophrenia-spectrum psychosis with insufficient response to standard antipsychotic treatment, monitored clozapine consideration may be clinically appropriate in selected adolescents, but causal conclusions from a single case must remain cautious.

## Patient perspective

Before I came to the hospital, I was really scared and confused. I felt like people were watching me and talking about me, and I could not fully trust my own family. At times, it felt like my thoughts and movements were not fully mine, like something else was controlling me. I also had trouble sleeping.

After starting treatment, I slowly began to feel calmer. The voices became less frequent, and my thoughts started to feel like my own again. Now I feel much better. I can trust my family, feel safer, and understand that what I went through was part of an illness.

## Data Availability

The original contributions presented in the study are included in the article/**Supplementary Material**. Further inquiries can be directed to the corresponding author.
