# Supplementary material for: Case Report: Severe adolescent-onset schizophrenia with Capgras syndrome and remission during clozapine-centered treatment after earthquake-related displacement and peer victimization
Source: Front Psychiatry. 2026 Jul 28;17:1887119. doi: 10.3389/fpsyt.2026.1887119 (PMC13457202; doi:10.3389/fpsyt.2026.1887119)
Supplement: Supplementary file 1 [file DataSheet1.docx]

CARE Checklist for Case Report - Revised after Associate Editor Minor Editorial Revisions

Manuscript ID 1887119 - Frontiers in Psychiatry

| **CARE topic** | **Item** | **Checklist item** | **Status** | **Manuscript location / correction made** |
| --- | --- | --- | --- | --- |
| Title | 1 | Diagnosis or intervention of primary focus followed by the words “case report”. | Addressed | Title begins with “Case Report:” and identifies adolescent-onset schizophrenia, Capgras syndrome, and clozapine-centered treatment. |
| Keywords | 2 | 2-5 key words identifying diagnoses or interventions, including “case report”. | Addressed | Frontiers allows 5-8 keywords; 8 keywords are provided and include “case report”. |
| Abstract | 3a-d | Uniqueness, main symptoms/findings, diagnosis/interventions/outcomes, and take-away lesson. | Addressed | Abstract uses cautious wording, avoids causal overstatement, and summarizes diagnostic complexity, treatment sequence, outcome, and take-away message. |
| Introduction | 4 | One or two paragraphs summarizing why the case is unique. | Addressed | Introduction updated with cautious novelty language: the contribution is framed as the clinical constellation within limited pediatric Capgras literature, not any single feature. |
| Patient information | 5a | De-identified patient-specific information. | Addressed | Patient information section states identifying details were omitted. |
| Patient information | 5b | Primary concerns and symptoms. | Addressed | Clinical Findings describes emergency presentation and symptom onset. |
| Patient information | 5c | Medical, family, and psychosocial history. | Addressed | Developmental, family, earthquake/displacement, peer-victimization, and school context included. |
| Patient information | 5d | Relevant past interventions with outcomes. | Partially addressed | No previous psychiatric admission was reported; previous outpatient treatment history was not fully available in the source material. |
| Clinical findings | 6 | Relevant physical examination and clinical findings. | Partially addressed | Mental status examination and neurological/medical work-up described; detailed physical examination/vital-sign data were not fully available in source material. |
| Timeline | 7 | Historical and current information organized as a timeline. | Addressed | Table 1 provides the clinical timeline from admission through 8-month follow-up; Table 3 now presents only selected key rating-scale points. |
| Diagnostic assessment | 8a | Diagnostic testing. | Addressed | Laboratory tests, MRI, EEG, substance screen, and neurology consultation described; Table 4 summarizes safety monitoring. |
| Diagnostic assessment | 8b | Diagnostic challenges. | Addressed | Diagnostic Assessment and Discussion explain overlap with bipolar, schizoaffective, trauma/dissociation, and organic causes. |
| Diagnostic assessment | 8c | Diagnosis and other diagnoses considered. | Addressed | Diagnostic Assessment and Table 2 provide structured differential diagnosis. |
| Diagnostic assessment | 8d | Prognosis where applicable. | Partially addressed | 8-month stability reported; longer-term prognosis requires continued follow-up. |
| Therapeutic intervention | 9a | Types of therapeutic intervention. | Addressed | Pharmacological interventions, cross-titration, and inpatient care described. |
| Therapeutic intervention | 9b | Administration of intervention: dosage, strength, duration. | Addressed | Medication doses and titration timeline included in text and Table 1. |
| Therapeutic intervention | 9c | Changes in intervention with rationale. | Addressed | Olanzapine addition, sertraline rationale, clozapine initiation, and cross-titration rationale clarified; clozapine rationale streamlined. |
| Follow-up and outcomes | 10a | Clinician- and patient-assessed outcomes. | Addressed | Selected key CGI-S, PANSS, and YMRS assessment points are summarized in simplified Table 3; patient perspective included. |
| Follow-up and outcomes | 10b | Follow-up diagnostic and test results. | Partially addressed | Clozapine safety values included; no additional 8-month structured rating-scale scores available, as stated in text/Table 3. |
| Follow-up and outcomes | 10c | Adherence and tolerability. | Partially addressed | Inpatient adherence/tolerability clinically monitored; formal outpatient adherence assessment details not fully available. |
| Follow-up and outcomes | 10d | Adverse and unanticipated events. | Addressed | Mild sedation/hypersalivation and absence of serious events reported; clozapine adverse effects discussed and monitored in Table 4. |
| Discussion | 11a | Strengths and limitations. | Addressed | Strengths and limitations retained in a more concise Discussion. |
| Discussion | 11b | Relevant medical literature. | Addressed | Relevant pediatric Capgras, first-episode psychosis, risk, treatment, and clozapine literature retained while repetition was reduced. |
| Discussion | 11c | Scientific rationale for conclusions and possible causes. | Addressed | Causality remains cautious; stress-diathesis formulation and alternative explanations for clozapine response are stated more concisely. |
| Discussion | 11d | Take-away lessons in a conclusion. | Addressed | Practical Take-Home Messages and cautious Conclusion retained; wording avoids overstating novelty or causality. |
| Patient perspective | 12 | Patient perspective on treatment received. | Addressed | Patient perspective retained without adding new unsupported wording. |
| Informed consent | 13 | Informed consent provided. | Addressed | Legal guardian consent and patient assent reported; documentation retained by authors. |
